# Life-threatening coma and full-thickness sunburn in a patient treated with transdermal fentanyl patches: a case report

**DOI:** 10.1186/1752-1947-6-220

**Published:** 2012-07-26

**Authors:** Katia Sindali, Katie Sherry, Sankhya Sen, Baljit Dheansa

**Affiliations:** 1 Queen Victoria Hospital, Holtye Road, East Grinstead, West Sussex, RH19 3DZ, United Kingdom

**Keywords:** Fentanyl, Opioid overdose, Sunburn

## Abstract

**Introduction:**

Fentanyl transdermal patches have been widely used in the treatment of chronic pain and in palliative care settings since 1991 in cases where prolonged opioid use is often necessary. Transdermal drug delivery is deemed safe and effective with the advantages of delivering a steady dose of the drug and improving patient compliance due to its ease of use. However, intentional and unintentional misuse and overdose using transdermal opioid patches has been widely reported in the literature.

**Case presentation:**

We describe the case of a 77-year-old Caucasian woman who developed severe opioid toxicity while sun tanning, likely due to altered fentanyl transdermal patch function in a heated environment. As a result of prolonged sun exposure due to an opioid-induced coma she then sustained hyperthermia and severe burns to her abdomen and lower limbs. This inadvertent fentanyl overdose necessitated initial treatment in intensive care and follow on care in a specialist burn unit.

**Conclusion:**

Patients who are using fentanyl patches and their relatives should be educated about how to use the patch safely. Healthcare practitioners should warn patients about the possibility of overdosing on transdermally delivered drugs if used incorrectly. They should avoid strenuous activities and external heat sources such as warming blankets, hot water bottles, saunas, hot tubs or sunbathing and should seek medical attention if they develop a fever. Additionally, any burns sustained in the context of altered consciousness levels such as in this case with opioid overdose should raise suspicion about a potential deeper burn injury than is usually observed.

## Introduction

We present the case of a 77-year-old Caucasian woman who showed classic signs of opioid overdose while using a fentanyl patch after prolonged sun exposure leading to coma. This was further complicated by full-thickness burns requiring transfer to and treatment in a specialist burn center.

The analgesic fentanyl is a synthetic phenylpiperidine derivative 50 to 100 times more potent than morphine. It was introduced into clinical practice in the1960s [1]. Several methods of administration exist including oral preparations, intravenous injections and the transdermal patch. Over the past few years, the Fentanyl Transdermal System has been increasingly used in the treatment of chronic pain and in palliative medicine.

Millions of patients have used a transdermal fentanyl patch to control their chronic pain since this medication delivery system was approved by the US Food and Drug Administration (FDA) in 1991.

One of the main advantages of transdermal drug delivery is the steady release of the substance resulting in a consistent plasma concentration. In addition, the weekly or twice weekly application is convenient and easy to use for patients, thus improving compliance.

However, numerous fentanyl-related mortalities and morbidities have been reported in the literature. Three case reports have described opioid overdose in patients after exposure of the patch to heat, either from an external source or through strenuous activity, resulting in increased core body temperature and subsequently increased release of the drug which led to an overdose and in one case, fatal respiratory arrest [2-4].

We believe that this is the first case report resulting in coma and full-thickness burns from the Fentanyl Transdremal System and highlights the importance of providing clear advice to patients to avoid heated environments in order to avoid this potentially life-threatening complication.

## Case presentation

A 77-year-old Caucasian woman who was using fentanyl patches (50 micrograms/hour) to alleviate chronic back pain fell asleep in the sun while on holiday in the South of France. Her only other co-morbidity was atrial fibrillation. She was found unconscious six hours later and an ambulance was called. Upon arrival of the medical team, the patient displayed signs of opioid overdose with respiratory depression, miosis and a Glasgow Coma Scale (GCS) of 3/15. Her core body temperature was 41°C. She was given two doses of naloxone promptly which increased her GCS to 5/15 (E1 V2 M2), and was started on intravenous paracetamol. She was intubated, ventilated and admitted to the intensive care unit of the local hospital. A full clinical examination revealed widespread erythema covering the majority of the anterior surface of her body as well as widespread blistering over her abdomen and on both lower limbs. She remained ventilated for 48 hours and required intravenous fluids resuscitation to correct her dehydration, acute kidney injury and electrolyte imbalance. She made a gradual recovery and was discharged seven days after admission with no neurological sequelae.

**Figure 1:**
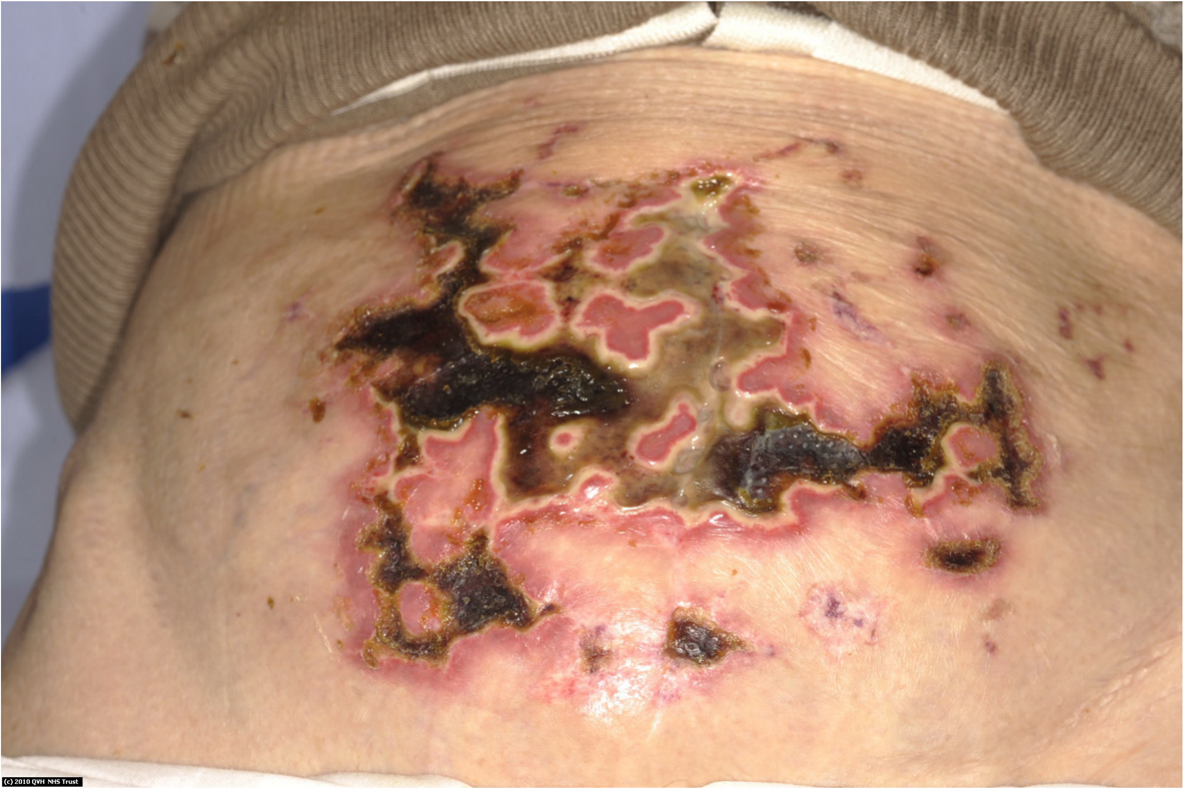
**Figure illustrating full**-**thickness burns on central abdomen needing surgical debridement.**

**Figure 2:**
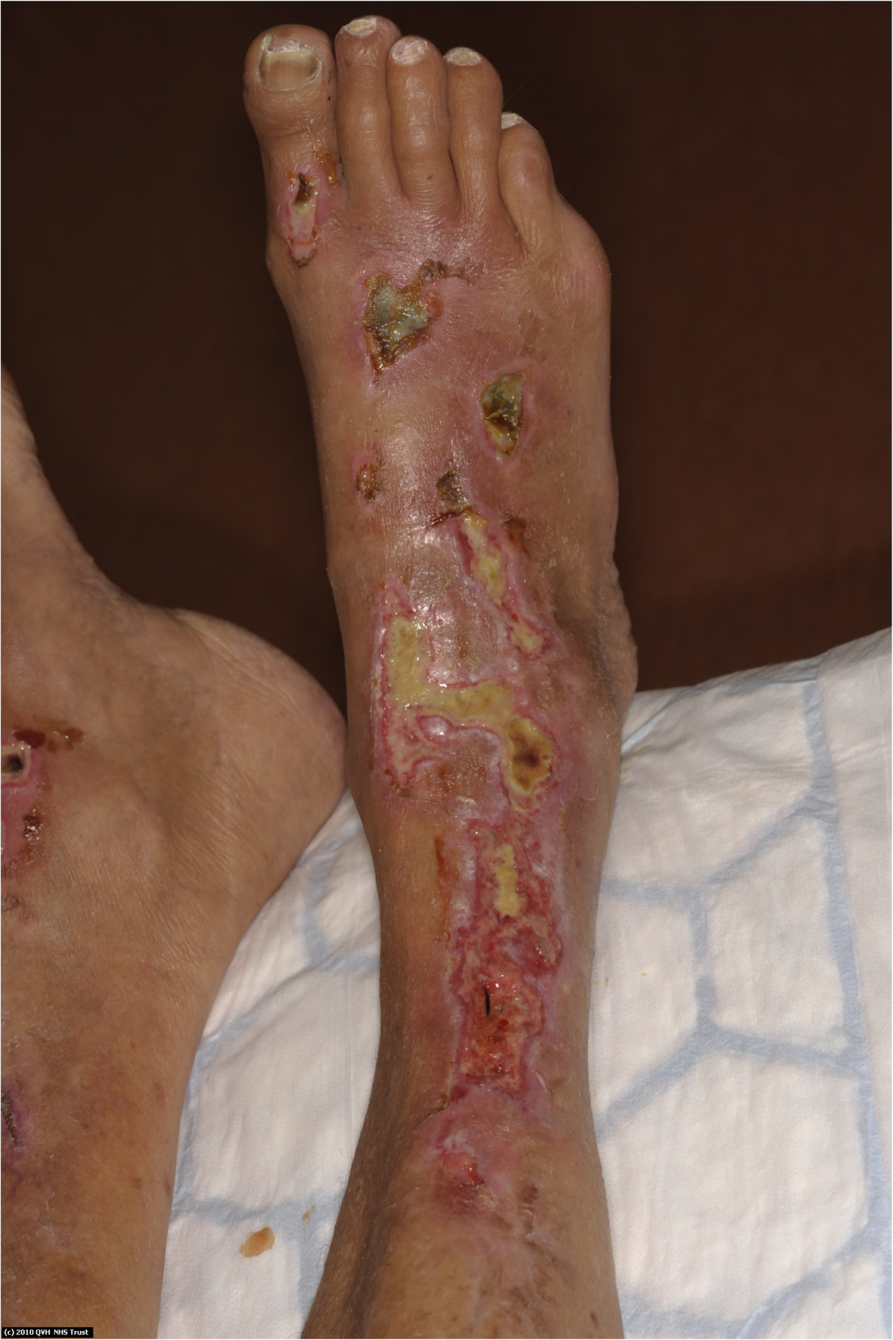
**Figure illustrating full**-**thickness burns on lower legs treated conservatively.**

Immediately after returning to the United Kingdom, she was referred to a specialist burn unit for further management of her burn wounds. She was found to have mixed-depth burns including full-thickness areas on her abdomen, both thighs and lower legs which were treated conservatively with Flamazine (silver sulfadiazine cream) (Figures 1 and 2). She required surgical debridement of her necrotic abdominal burn wound which subsequently healed well along with the other wounds.

## Discussion

This case highlights a potentially very serious side effect of transdermal fentanyl. Our patient nearly died as a result of this inadvertent fentanyl overdose causing coma, respiratory depression and severe dehydration with deep dermal burns from sun exposure.

Heat is known to increase skin permeability to drugs by several mechanisms. Raised temperatures increase microcirculation and blood vessel permeability, which aids drug transfer into the systemic circulation [5].

It is estimated that an increase in normal skin temperature from 32°C to 40°C results in a 10- to 15-fold increase in cutaneous blood flow as demonstrated by laser Doppler [6] and that a 3°C increase in body temperature elevates the peak fentanyl plasma concentration by 25% [7]. Another study showed that heating the patch during the first four hours after application increased the maximum plasma concentration almost three fold [8].

Three other cases of fentanyl overdose directly attributable to heat have been reported. These include one patient who inadvertently placed a heating pad over their fentanyl patch and subsequently developed symptoms of opioid overdose. In another patient, a warming blanket was placed over their fentanyl patch during surgery and they also developed signs of opioid overdose. In a third case, excess outdoor activity on a sunny day also led to coma in a patient using a fentanyl patch [2-4].

In this case, the patient’s core body temperature was 41°C when she was found unconscious and, although the plasma concentration of fentanyl was not measured at the time of hospitalization, one can assume that the heated environment caused a rise in the fentanyl plasma concentration, leading to respiratory depression, altered consciousness levels and eventually coma. Her comatose state inhibited her natural waking mechanism causing hyperthermia and unusually severe sunburn requiring treatment in a specialist burn unit.

## Conclusion

Patients who are using fentanyl patches and their relatives should be educated about how to use the patch safely. Healthcare practitioners should warn patients about the possibility of overdosing on transdermally delivered drugs if used incorrectly. They should avoid strenuous activities and external heat sources such as warming blankets, hot water bottles, saunas, hot tubs or sunbathing and should seek medical attention if they develop a fever.

Finally, any burns sustained in the context of altered consciousness levels such as in this case with an opioid overdose should raise suspicions about a potential deeper burn injury than is usually observed.

## Consent

Written informed consent was obtained from the patient for publication of this case report and any accompanying images. A copy of the written consent is available for review by the Editor-in-Chief of this journal.

## Competing interests

The authors declare that they have no competing interests.

## Authors’ contributions

KS, KS and SS prepared the manuscript. BD gave final approval for publication of this article. All authors read and approved the final manuscript.
